# CRF1-R Activation of the Dynorphin/Kappa Opioid System in the Mouse Basolateral Amygdala Mediates Anxiety-Like Behavior

**DOI:** 10.1371/journal.pone.0008528

**Published:** 2009-12-31

**Authors:** Michael R. Bruchas, Benjamin B. Land, Julia C. Lemos, Charles Chavkin

**Affiliations:** 1 Department of Pharmacology, University of Washington, Seattle, Washington, United States of America; 2 Graduate Program in Neurobiology and Behavior, University of Washington, Seattle, Washington, United States of America; Max-Planck-Institut für Neurobiologie, Germany

## Abstract

Stress is a complex human experience and having both rewarding and aversive motivational properties. The adverse effects of stress are well documented, yet many of underlying mechanisms remain unclear and controversial. Here we report that the anxiogenic properties of stress are encoded by the endogenous opioid peptide dynorphin acting in the basolateral amygdala. Using pharmacological and genetic approaches, we found that the anxiogenic-like effects of Corticotropin Releasing Factor (CRF) were triggered by CRF_1_-R activation of the dynorphin/kappa opioid receptor (KOR) system. Central CRF administration significantly reduced the percent open-arm time in the elevated plus maze (EPM). The reduction in open-arm time was blocked by pretreatment with the KOR antagonist norbinaltorphimine (norBNI), and was not evident in mice lacking the endogenous KOR ligand dynorphin. The CRF_1_-R agonist stressin 1 also significantly reduced open-arm time in the EPM, and this decrease was blocked by norBNI. In contrast, the selective CRF_2_-R agonist urocortin III did not affect open arm time, and mice lacking CRF_2_-R still showed an increase in anxiety-like behavior in response to CRF injection. However, CRF_2_-R knockout animals did not develop CRF conditioned place aversion, suggesting that CRF_1_-R activation may mediate anxiety and CRF_2_-R may encode aversion. Using a phosphoselective antibody (KORp) to identify sites of dynorphin action, we found that CRF increased KORp-immunoreactivity in the basolateral amygdala (BLA) of wildtype, but not in mice pretreated with the selective CRF_1_-R antagonist, antalarmin. Consistent with the concept that acute stress or CRF injection-induced anxiety was mediated by dynorphin release in the BLA, local injection of norBNI blocked the stress or CRF-induced increase in anxiety-like behavior; whereas norBNI injection in a nearby thalamic nucleus did not. The intersection of stress-induced CRF and the dynorphin/KOR system in the BLA was surprising, and these results suggest that CRF and dynorphin/KOR systems may coordinate stress-induced anxiety behaviors and aversive behaviors via different mechanisms.

## Introduction

Stress is a complex human experience and has both rewarding as well as aversive motivational properties. The adverse effects of stress are well documented, yet many of the underlying mechanisms remain unclear and controversial. Stress mobilizes the corticotropin releasing factor (CRF)/urocortin neuropeptide systems to activate the hypothalamic-pituitary- adrenal axis (HPA), and extra-hypothalamic actions of CRF can stimulate the neuronal circuits responsible for stress-induced anxiety, dysphoria, and reinstatement of drug abuse behaviors [1]–[8]. In rodent models of motivated behavior, CRF produces conditioned place aversion (CPA) [1], [7], and in some cases CRF receptor antagonists have antidepressant effects in human studies [9]. Other groups have demonstrated that CRF has anxiogenic properties [10]–[12], and it has been posited that these effects of CRF initiate stress-induced relapse to drug seeking and drug withdrawal behaviors [3], [4], [6], [13], [14].

CRF acts directly on neurons in key regions of the central nervous system (e.g. bed nucleus of the stria terminalis and amygdalar complex) to coordinate the behavioral stress response through the activation of two receptor subtypes [15]. The CRF system is comprised of the neuropeptides CRF and urocortin I that bind to and activate both CRF_1_-receptors (CRF_1_-R) and CRF_2_-receptors; whereas the peptides urocortin II and urocortin III are more selective CRF_2_-receptor agonists [15], [16]. CRF_1_-R activity is thought to be prodepressant and anxiogenic [10], [11], [15], whereas CRF_2_-R activity has been suggested to have a complementary stress coping role. However, the processes by which CRF and the urocortins mediate stress-induced behaviors through their two receptor systems are still under active investigation.

Recent work has shown that the aversive effects of stress can be mediated by CRF-induced activation of type 2 CRF receptors (CRF_2_-R) and subsequent activation of kappa opioid receptors (KOR) by dynorphin [1]. The dynorphin/KOR system is a critical mediator in stress-induced depression and stress-induced reinstatement of drug-seeking [8], [17]–[21]. These previous studies provided new insight into a potential connection between the dynorphin/KOR and stress-CRF systems. It also suggested that the dysphoria-mediated by these systems may in fact be caused by an initial anxiety-like response during the CRF or stress experience. Interestingly, a possible role for stress-induced dynorphin release in anxiety-like behavior has been suggested [22], but a relationship to CRF has not been established. In this study, we dissected the linkage between the CRF and dynorphin systems in the brain, and studied how these interactions may mediate anxiety-like behaviors.

## Materials and Methods

### Animals

Male C57Bl/6 mice (Charles River) weighing 22–30 g were used. Prodynorphin (*Pdyn)* gene deletion on C57Bl/6 background were generated as previously described [1]. CRF_2_-R(−/−) and CRF_1_-R(−/−) mice were gifts of Antonello Bonci and Wylie Vale, and were backcrossed to a C57Bl/6 background for ≥5 generations in our laboratory (in addition to ∼5 generations in the previous laboratory). Pair-wise knockout and littermate control wild type mice were used throughout the experimental studies described. We also note that the viability of CRF_1_-R(−/−) mice on C57Bl/6 background was very poor; heterozygote crosses produced significantly fewer homozygous CRF_1_-R (−/−) male mice than predicted (p<0.05, chi square, data not shown). After 1 year of breeding, only 2 of these mice survived stereotaxic surgery and were used, and only for qualitative anatomical analysis. All animal protocols were approved by the UW Institutional Animal Care and Use Committee.

### Drugs and Chemicals

Rat/human corticotropin releasing factor (CRF), norbinaltorphimine (norBNI)-HCl, cyclo (31–34)[D-Phe^12^,Nle^21,38^,Glu^31^, Lys^34^]Ac-hCRF (4–41) (stressin 1), and (±)U50,488 were from Tocris Bioscience (Ellisville, MO). Additional U50,488 and norBNI were provided by the NIDA Drug supply program. Mouse urocortin III was from Phoenix Pharmaceuticals (Belmont, CA). Peptides were dissolved in saline/0.05% acetic acid. U50,488 and norBNI were dissolved in saline. Drugs were administered at 10 ml/kg (i.p.) or 2 µl/animal (i.c.v).

### Intracerebroventricular (i.c.v.) Cannulation and Local norBNI Injections

Mice were cannulated and injected i.c.v. as previously described [1]. Following surgery mice recovered for 6–8 days. Mice were slowly injected (2 min) with saline or peptide (CRF, urocortin III, or stressin I, 2 µl, i.c.v.), then either placed in the testing chamber for place aversion studies or back in their home cage in the darkened elevated plus maze testing room for 30 min prior to EPM testing. One week prior to i.c.v. injection of peptide, some mice were also anesthetized and injected bilaterally in the BLA (lateral ±3.1 mm, posterior −1.8 mm, and 5 mm depth (from bregma) or VPN (ventral posterior thalamic nucleus) (lateral ±1.37, posterior −1.82, and 4.35 depth) with norBNI or vehicle at 100 nl/min (10 min), using a Hamilton syringe (1 µl, 12.5° beveled tip) adapted from Shirayama et al [23]. The needle was removed 3–5 min after injection. Because norBNI is a long-lasting antagonist and blocks KOR for up to 3 weeks *in vivo* [24], [25], we were able to wait 1 week after the injection to allow animals to recover from injection and surgery prior to EPM testing. After behavioral testing, sites of local norBNI injection and receptor blockade were confirmed by measuring KORp-ir induced by 10 mg/kg U50,488 given 30 min prior to perfusion. We did not observe any lesions or gliosis at any of the injection sites, and the needle track was no longer visible 2 weeks after injection. We also note that due to low birth rate (see above), and low survival rate of CRF_1_-R (−/−) male mice (20%) following i.c.v. cannulation surgery (24 hrs to 1 week), as compared to >95% surgery survival for all other wild type and knockout groups, we were unable to generate sufficient age-matched controls to successfully use this group in our behavioral studies.

### Elevated Plus Maze (EPM) Testing

EPM testing was performed in a sound attenuated room with dark brown walls and black ceiling, maintained at 23°C. For all studies (except supplemental figure 1, which was performed at 200 lux), lighting was 50 lux, and performed in the afternoon between 13:00–1600 hrs. This procedure is widely accepted to have predictive validity based on responses to anxiolytic drugs [26], [27]. The EPM (Med Associates, St Albans, VT) was made of black plastic (dimensions: 38 cm Open Arm length×7.62 cm Width×74 cm Height, with a 0.5 cm lip on each open arm) and was cleaned with 70% ethanol between trials. For testing, mice were placed in the center sector of the maze facing the open arm and allowed to roam freely for 6 min. Movements were video-recorded and analyzed using Ethovision (Noldus, Netherlands). We used open or closed arm times expressed as percentages total time as our primary measures of anxiety-like behaviors. The observed locomotor activity in the EPM was not different between groups, because total arm entry estimates (21±6) were not statistically different between groups, suggesting that mice freely roamed the maze. Furthermore, all drug treatments/injections occurred 30 min prior to placing animals in the maze, a time that is well after the peak hypolocomotor effects of KOR agonism.

**Figure 1:**
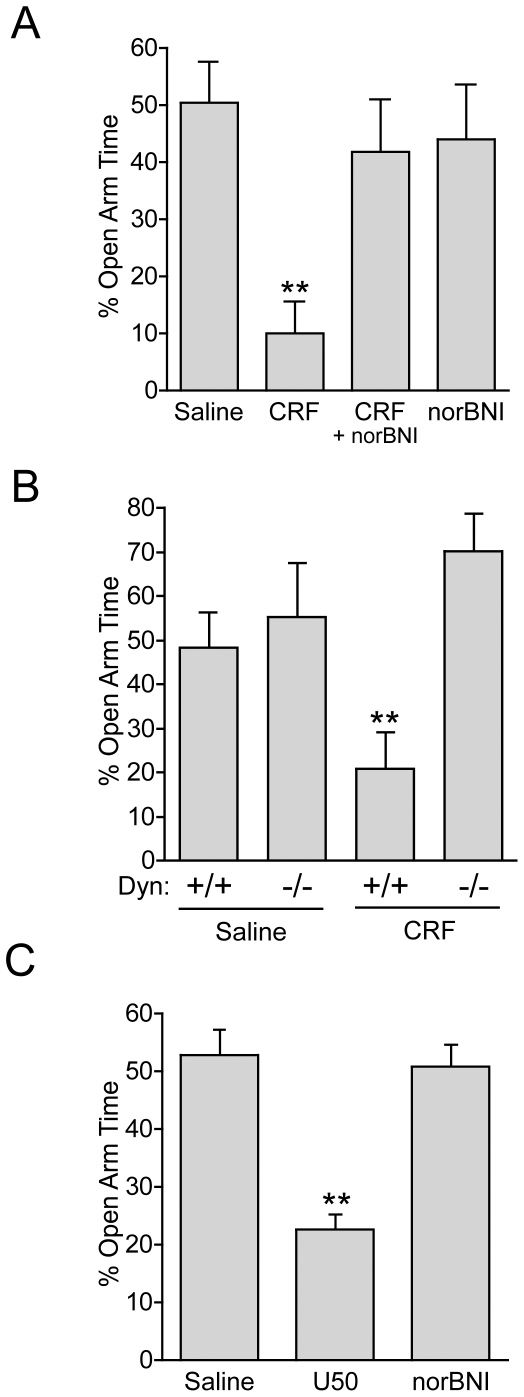
CRF-induced anxiety-like behavior is mediated by the dynorphin/KOR system. ***A***, CRF injection (1 µg, i.c.v., 30 min prior to assay) produced a significant anxiety-like effect (decrease in % open arm time) in the elevated plus maze (EPM) compared with saline injected controls. The effect of CRF was blocked by pretreatment with the KOR antagonist norBNI (10 g/kg, i.p., 2.5 hrs prior to test) (n = 7–8; two-way ANOVA; main effect of CRF, F_(1,24)_ = 6.323, *p*<0.05; interaction of CRF and pretreatment, F_(1,24)_ = 7.897 *p*<0.05; ** *p*<0.01 Bonferroni *post hoc*)***B***, CRF (1 µg, i.c.v., 30 min prior to assay) produced a significant anxiety-like effect in wildtype littermate control Dyn(+/+) mice, but had no effect in *prodynorphin* knockout animals (Dyn −/−) (n = 4–8 per group, *p*<0.01 CRF in Dyn +/+ vs Dyn −/−). CRF also caused a significant increase in open arm time in Dyn (−/−) animals (*p*<0.01, *t*-test, open arm vs. closed arm time, data not shown) ***C***, Activation of KOR by the selective KOR agonist U50,488 (U50, 5 mg/kg, i.p.) was also sufficient to cause significant anxiety-like behavioral effects, whereas treatment with norBNI alone had no effect on the percent open arm or closed arm time in the EPM under these conditions. (n = 8, one-way ANOVA, **p<0.01, U50 vs. saline).

Acute stress-induced EPM behavior following local drug or vehicle injection into BLA, was measured following a modified acute swim stress exposure [28]. Mice were placed for 5 min in a 5 L beaker (40 cm tall×25 cm in diameter) filled with 3.5 L of 30°C water, carefully dried after removal, and then placed back in the home cage for 30 min in order to mimic the time between i.c.v. CRF injection and testing described above. Mice were then tested for time spent in the open and closed arms of the EPM.

### Conditioned Place Aversion

Cannulated mice were trained in a balanced, three-compartment conditioning apparatus as described [1]. Lack of compartment bias was confirmed in this study by the experimental results (described below). Briefly, mice were pretested in the apparatus, and time spent in each compartment during the 30 min session was video recorded and analyzed using Ethovision (Noldus). Mice were randomly assigned drug and saline training compartments in a counterbalanced experimental design, and received saline (2 µL, i.c.v.) in the mornings and CRF (1 µg/2 µL i.c.v.) in the afternoons of days 2 and 3. Aversion was assessed on day 4 by recording time spent in each of the 3 compartments during a 30 min post-training session. Preference or aversion scores are presented as the time each individual animal spent in the drug paired side in the post-test minus the time spent in the drug-paired side during the pre-test.

### Immunohistochemistry

Procedures to assess KORp-ir were previously described [1], [29]. Cannulated mice were i.c.v. injected with CRF or saline, then 30 min later were anesthetized with isoflurane (Sigma) and intracardially perfused with 4% paraformaldehyde in phosphate buffer (PB) (0.1 M sodium phosphate, pH 7.4). For those animals that had been locally injected with norBNI, mice were injected with U50,488 (10 mg/kg, i.p.) 30 min prior to cardio-perfusion. Brains were dissected and cryoprotected with solution of 30% (w/v) sucrose in PB at 4°C overnight, cut into 40 µm sections, and placed in PB until processing. Affinity purified rabbit anti-phospho-KOR (KORp) antibody (1∶50 dilution) [30] diluted in blocking buffer: PBS containing 0.3% triton X-100 and 5% normal goat or donkey sera, was used at 15–30 µg/section. Sections were subsequently washed with PBS then incubated with anti-Rabbit IgG Alexa Fluor 488, 555 or 633 (1∶500, Molecular Probes, Eugene, OR). Positive KORp-immunoreactive (ir) cells were quantified from 2–4 separate BLA slices taken from 2–4 animals (fields of 600 µm^2^) that were assigned letters and counted by investigators blind to treatment group. A cell designated as positive for KORp-ir if optical density was above a standardized background threshold value for each treatment group, as determined by using Metamorph software (Dovingtown, PA).

### Data Analysis

Data are expressed as means +/− SEM. For experiments having a 2×2 design, two-way ANOVAs were used, followed by Bonferroni *post-hoc* tests if significant (*p*<0.05) main or interaction effects were found. All other experiments used *t* tests or one-way ANOVA followed by Bonferroni *post-hoc* if the main effect was significant at *p*<0.05. Statistical analyses were conducted using GraphPad Prism (4.0) (GraphPad, San Diego, CA) or SPSS (Version 11.0, SPSS, Chicago, IL).

## Results

### CRF-Induced Anxiety-Like Behavior Is Mediated by the Dynorphin/KOR System

The elevated-plus maze (EPM) was used to measure exploratory behaviors under dimly illuminated, less stressful conditions that produced nearly equal times in the open and closed arms to best reveal anxiogenic effects of treatment. Mice receiving 1 µg CRF (i.c.v.) spent significantly less time in the open arms of the EPM compared to saline-injected controls (Fig. 1A). Surprisingly, the CRF-induced decrease in open arm time was blocked by pretreatment with the KOR-selective antagonist norbinaltorphimine (norBNI) (10 mg/kg, i.p.) (n = 7–8; two-way ANOVA; main effect of CRF, F_(1,24)_ = 6.323, *p*<0.05; interaction of CRF and pretreatment, F_(1,24)_ = 7.897 *p*<0.05; Bonferroni *post hoc* CRF vs. CRF + norBNI). These results indicate that the anxiogenic-like effects of CRF require activation of the dynorphin/KOR system. *Pdyn (−/−*) mice lacking a functional prodynorphin gene [31] injected with saline behaved identically to wildtype, littermate *Pdyn (+/+)* mice in the EPM, suggesting that dynorphin does not control the basal anxiety state in the absence of stress (Fig. 1B). In contrast, CRF did not significantly affect open arm time in *Pdyn (−/−)* mice (n = 4–8, two-way ANOVA; main effect of genotype, F_(1,20)_ = 8.47, *p*<0.01; interaction RF and genotype, F_(1,20)_ = 4.83, *p*<0.05; Bonferroni post hoc CRF *Pdyn −/−* vs. CRF *PDyn +/+*). Interestingly *Pdyn (−/−)* mice spent significantly more time in the open arm than the closed arm after CRF treatment (*p*<0.01, paired *t* test, closed arm vs. open arm), implying that in animals lacking dynorphin, CRF may promote exploratory behavior. These observations suggest that CRF-induced anxiety-like responses require dynorphin expression.

Mice injected with the selective KOR-agonist U50,488H (5 mg/kg, i.p.) spent significantly less time in the open arm than their matched saline-injected controls (Fig. 1c, n = 5–7, one-way ANOVA, F_(2,17)_ = 29.87; *p*<0.001, Saline vs. U50; Bonferroni *post-hoc*), implying that KOR activation was sufficient to produce anxiety-like behaviors. Similar to cannulated mice (Fig. 1A), norBNI (10 mg/kg, i.p., 2.5 hrs before assay) treatment in the absence of CRF or U50,488H administration, did not affect open arm time (Fig. 1C), which corroborates the lack of effect of *pdyn (−/−*) in this assay. Together these results demonstrate that basal dynorphin ”tone“ is negligible in dim light and that the anxiogenic-like effects of CRF require activation of the dynorphin/kappa opioid system.

We also measured the effect of disruption of the dynorphin/KOR system under intrinsically stressful, ”bright light“ conditions (see methods). Saline-treated mice spent significantly (n = 4–6; *p*<0.05; *t_6_* = 2.917) less time in the open arm than their norBNI pretreated (10 mg/kg, i.p) counterparts. Furthermore, *Pdyn* (−/−) knockout mice also showed a significantly greater open arm time compared to wild type controls (n = 5; p<0.05, *t_7_* = 3.147) (Figure S1). These data corroborate other reports [22], [32] using similar assay conditions and strongly support a role for dynorphin/KOR in mediating anxiety-like behaviors.

### CRF-Induced Anxiety-Like Behavior Is Mediated by CRF_1_-R but Not CRF_2_-R Activation

**Figure 2:**
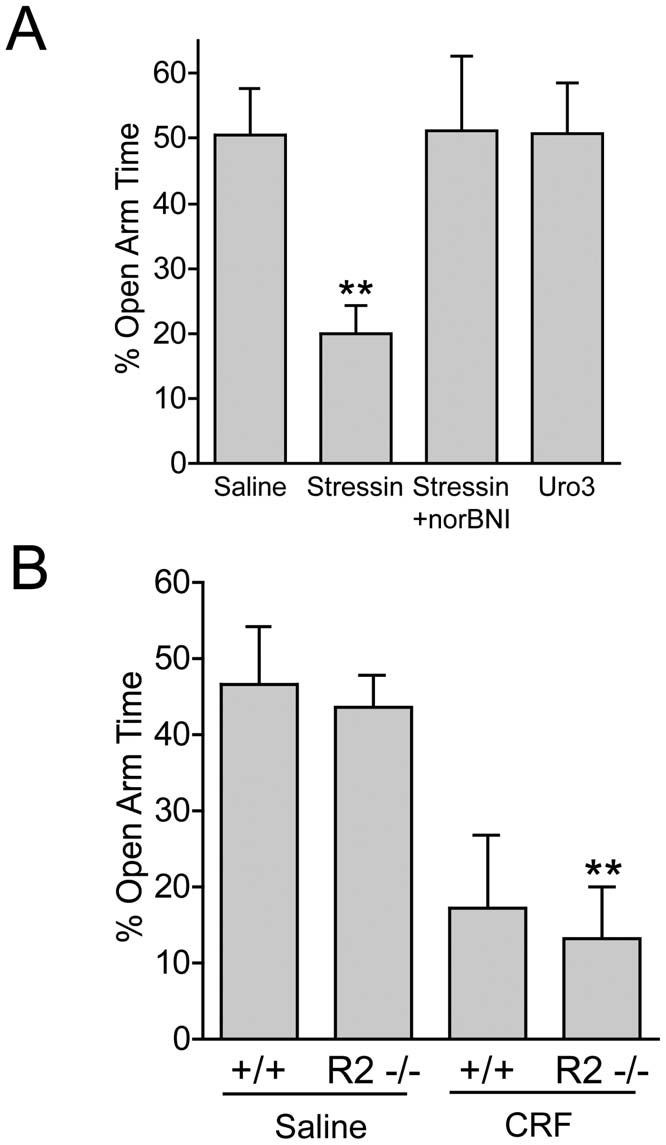
CRF-induced anxiety-like behavior is mediated by CRF_1_-R but not CRF_2_-R activation. ***A***, Administration of stressin 1 (0.5 µg, i.c.v.), a selective CRF_1_-R agonist, produced significant anxiety-like behavioral effects. Pretreatment with the KOR antagonist norBNI (10 mg/kg, i.p., 2.5 hrs prior to test) blocked the stressin1-induced (Stsn) decrease in percent open arm time (n = 6–8, one-way ANOVA, F_(3, 20)_ = 4.658; **p<0.05, Saline vs. Stressin 1). In contrast, the EPM scores for wildtype mice injected with the CRF_2_-R selective agonist urocortin III (Uro3) (0.5 µg, i.c.v.), were not significantly different from the saline-treated group (n = 6–8). ***B***, Consistent with the above results, CRF administration to CRF_2_-R (−/−) mice still produced significant anxiety-like behavior (n =  4–8, two-way ANOVA; main effect of CRF, F_(1, 21)_ = 19.02, *p*<0.001; no main effect of genotype, F_(1,22)_ = 0.26, *p*>0.05; Bonferroni post-hoc, ** p<0.01 saline (−/−) vs. CRF (−/−)).

Prior studies showed that CRF_2_-R activation caused dynorphin-dependent conditioned place aversion (CPA) [1]; however, the relative contributions of CRF_1_-R and CRF_2_-R subtypes in dynorphin/KOR-dependent anxiety-like behaviors are unknown. The CRF_1_-R-selective agonist stressin 1 [33] (0.5 µg, i.c.v.) significantly decreased percent open arm time in the EPM (Fig. 2A) (n =  6–8, one-way ANOVA, F_(3, 20)_ = 4.658; *p*<0.05, Saline vs. Stressin 1). Consistent with the findings described above, the anxiogenic-like effects of stressin 1 were significantly blocked by norBNI (Fig. 2A) (*p*<0.05, Saline vs. Stressin + norBNI), as the percent open arm time was equal to saline-treated animals. Additionally, mice injected with the CRF_2_-R-selective agonist urocortin III [34] (0.5 µg, i.c.v.) showed no difference from vehicle-treated controls in percent time spent in the open arm (Fig. 2A). Using CRF_2_-R knockout mice [11] (CRF_2_-R −/−) and wildtype (+/+) littermate controls to corroborate these results, we found that the effects CRF in the EPM were unaffected by deletion of the *CRF_2_-R* gene (Fig. 2*B*, n =  4–8, two-way ANOVA; main effect of CRF, F_(1, 21)_ = 19.02, *p*<0.001; no main effect of genotype, F_(1,22)_ = 0.26, *p*>0.05; Bonferroni *post-hoc*). Together these data support the conclusion, that CRF_1_-R activation mediates CRF-induced dynorphin/KOR-dependent anxiety-like behavior.

### CRF-Induced Conditioned Place Aversion Is CRF_2_-R Dependent

**Figure 3:**
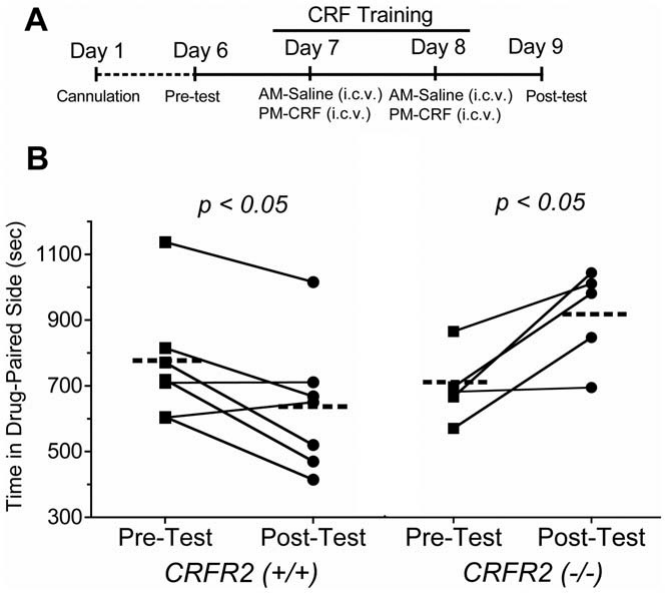
CRF-induced conditioned place aversion is CRF_2_-R dependent. ***A***, Schematic of the conditioning procedure used to assay aversion. ***B***, Conditioned place aversion expressed as the individual animal pre-test (squares) connected by line to the same animal's post-test (circles) times spent in the CRF-paired compartment. Dashed lines represent the mean times during either the pre-test or post-test for each group. There was no significant difference between CRF_2_-R (+/+) and CRF_2_-R(−/−) mice in pretest scores. CRF (1 µg, i.c.v.) induced a significant place aversion in CRF_2_-R +/+ mice (post-test minus pre-test in the drug paired side, seconds) of -129±43 sec. In contrast, CRF injected in CRF_2_-R(−/−) mice induced a significant place preference (*p*<0.05) of 235±68 sec (n = 5–7, p<0.05 pre-test minus post-test for both groups).

Our prior pharmacological characterization [1] led us to predict that CRF_2_-R receptors are required for CRF-induced aversion, and this current report suggests that CRF-induced anxiety-like behavior may be mediated by a different CRF_1_-R dependent activation of dynorphin/KOR signaling. In order to build on this concept using a conditioned place aversion paradigm (Fig. 3*A*) where mice learn to associate CRF injection with a distinct environmental context, the aversive effects of CRF injection were examined in *CRF_2_-R* (−/−) mice (Fig. 3*B*). Wildtype littermate mice injected with CRF showed a significant place aversion (mean difference: -129±43 sec; 95% CI −238 to −22). In contrast to wild type, CRF_2_-R (−/−) mice showed a significant place preference (mean difference: +235±68 sec; 95% CI 46 to 425), not aversion (n = 5–7, P<0.05, paired t-tests). The basis for the preference to CRF in *CRF2-R (*−/−*)* mice is not clear, but it may be a consequence of the rewarding effects of dopamine release caused by CRF activation of CRF_1_-R expressing neurons in the ventral tegmental area [5], [35]. These data illustrate that CRF_1_R and CRF_2_R activation can initiate distinct dynorphin/KOR-dependent behaviors with CRF_1_R selectively initiating dynorphin-dependent anxiety-like responses and CRF_2_R initiating dynorphin-dependent place aversion.

### Stress-Induced Anxiety-Like Behavior Is Mediated by Dynorphin/KOR in the BLA

**Figure 4:**
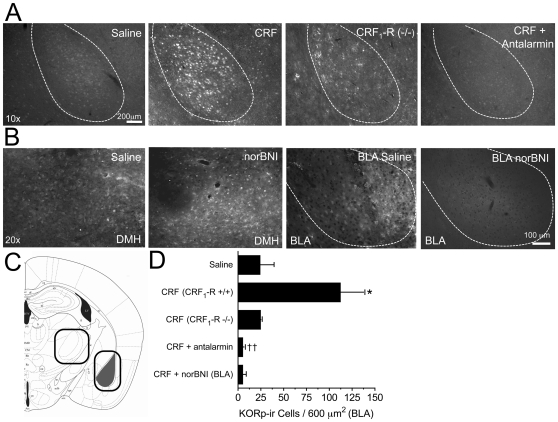
CRF-induces CRF_1_-R-dependent dynorphin/KOR activation in the BLA. ***A***, CRF (1 µg, i.c.v., 30 min) administration in wildtype mice increased in phospho-KOR-ir in the BLA (upper row, second panel). Pretreatment of wild type mice with antalarmin (10 mg/kg, i.p., injected 1hr before CRF) blocked the increased CRF-induced KORp-ir in the BLA (upper row, right panel). Similarly, KORp-ir did not qualitatively increase in CRF_1_-R (−/−) mice following CRF challenge (panel 3, top row, n = 2). As expected, mice injected with saline (vehicle, i.c.v.) showed low levels of KORp-ir in the BLA (top left). ***B*** *,* Local infusion of norBNI into the BLA of wildtype animals reduced the CRF-induced KORp-ir in the BLA (far right panel) but had no effect on CRF-induced KORp-ir the dorsal medial hypothalamus (DMH), a nearby brain structure with comparable levels KORp-ir in the untreated animal. This control region allowed for a regional confirmation of the sphere of norBNI blockade of KOR in injected animals. As predicted norBNI into the BLA had no effect on KORp-ir in the DMH. ***C***, Schematic of the area imaged and injected with local norBNI; the right box outlines the region imaged including the BLA and the left box outlines the dorsal medial hypothalamus (DMH), the closest adjacent brain structure in the same slice where there is high expression of KOR and KORp-ir was visualized following CRF-injection. ***D***, Quantification (per 600 µm^2^) of BLA KORp-ir following saline or CRF-injection. CRF induced a significant increase in KORp-ir as compared to saline, or antalarmin-treated groups. In animals locally injected with norBNI into the BLA, CRF-induced KORp-ir cell staining was not significantly different than saline treated mice. (*p<0.05, CRF vs. saline, †† *p*<0.01 CRF vs. CRF + antalarmin). Data are from 2–4 independent experiments.

The anxiogenic-like effects of CRF and stressin 1 suggest that CRF_1_-R receptor activation induces dynorphin release in the basolateral amygdala (BLA), a brain region regarded as necessary for anxiety-like behaviors [36]. Using a phosphoselective antibody (KORp) shown to selectively label sites of dynorphin action [1], [29], [30], we found that CRF injection significantly increased KORp-ir in the BLA (Fig. 4*A,C*) (n =  4, one-way ANOVA, F_(2, 10)_ = 10.66; Bonferoni post-hoc, *p*<0.05 vs. CRF vs. saline). Antagonism of CRF_1_-R with a selective dose of antalarmin (10 mg/kg, i.p.) [1] blocked the CRF-induced increase KORp-ir in the BLA (Fig. 4*A,D*) (one way ANOVA, F_(2, 10)_ = 10.66, *p*<0.01 CRF vs. CRF + antalarmin). Furthermore, *CRF_1_-R* (−/−) mice showed no marked CRF-induced increase in KORp-ir (Fig. 4*A*, n = 2).

To determine the behavioral consequences of CRF-induced dynorphin release and subsequent KOR activation, we injected saline or norBNI bilaterally into the BLA (2.5 µg/side) as described [21], [23]. We then measured CRF-induced EPM behavior and changes in KORp-ir. NorBNI antagonism of KOR is long-lasting and can persist for 2–3 weeks following a single injection [24], [25]. Control brain sections from mice stereotaxically injected with saline in the BLA show increased KORp-ir induced by CRF (i.c.v) (Fig. 4*B*). In contrast, KORp-ir was not increased in BLA from mice that had been stereotaxically injected norBNI in the BLA (Fig. 4*B,D*). The effects of norBNI injected in BLA were spatially restricted as shown by the presence of KORp-ir (80 KORp-ir cells/600 µm^2^) in the nearby dorsal medial hypothalamus (DMH) following BLA norBNI. The DMH is a ventral structure that also expresses high levels of KOR (Fig. 4*A*).

**Figure 5:**
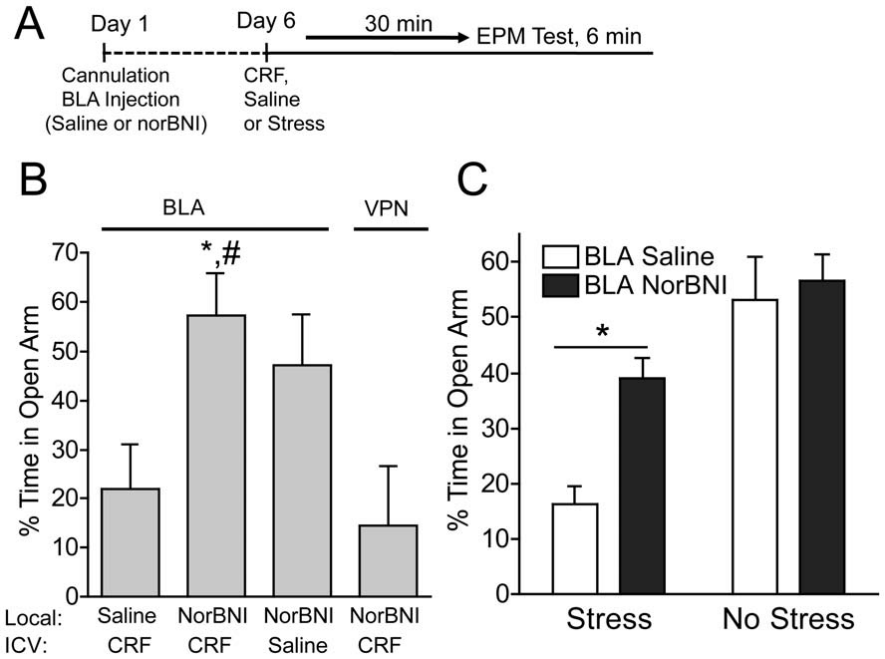
Stress and CRF-induced anxiety is mediated by dynorphin/KOR in the BLA. ***A***, Mice injected with saline (1 µl) into the BLA showed a decrease in percent open arm time behavior in the EPM following subsequent CRF (i.c.v) injection, whereas bilateral infusion of norBNI (2.5 µg/side, see methods) in the BLA significantly blocked the CRF-induced (1 µg, i.c.v.) decrease in percent open arm response. Control injection of norBNI into a nearby brain structure, the ventral posteromedial thalamic nucleus (VPN), had no effect on the CRF-induced anxiety-like behavior evident in the EPM. (*p<0.05, CRF-injected norBNI/BLA vs. CRF-injected saline/BLA; † *p*<0.05, CRF-injected norBNI/BLA vs. CRF-injected norBNI/VPN; n = 6–8 per group). ***B***, In further support of a CRF-dependent dynorphin/KOR interaction in the BLA, mice were exposed to a single acute swim stress (5 min), placed back in their home cage for 30 min and tested in the EPM. As expected, swim stress induced a significant decrease in the percent time in the open arm as compared to locally injected non-stressed control animals. The anxiogenic-like response to acute stress was significantly blocked in mice injected with norBNI in the BLA (Fig. 5*B*) (n = 5–6, F_(1, 16)_ = two-way ANOVA; main effect of stress, F _(1,16)_ = 23.54; main effect of treatment, F _(1,16)_ = 5.387; *p*<0.05, Bonferroni *post-hoc*).

In mice bilaterally injected with saline in BLA and subsequently CRF-treated had a percent open arm time comparable to other CRF-injected groups in this study (Fig. 5*B*). However, the anxiogenic-like response to CRF was abolished for mice injected with norBNI in the BLA (Fig. 5*B*) (n = 6–8, F_(2, 19)_ = 6.350, P<0.05, saline vs. norBNI BLA and norBNI VPN vs. norBNI BLA). In contrast, bilateral injection of norBNI in the ventral posteromedial thalamic nucleus (VPN), a structure that is contiguous and just medial to the BLA and also expresses KOR (Figure S2), had no affect on the CRF-induced decrease in percent time in the open arm (Fig. 5*B*). Together, these data suggest a previously unrecognized connection between BLA-dependent stress-induced anxiety-like behavior, stimulation of the CRF_1_-R, and activation of the dynorphin/KOR system.

In order to examine the whether the effects of CRF in BLA on KOR-mediated anxiety-like behavior generalize to other environmental stressors, we adapted a published procedure using a single episode of swim stress [28], then measured EPM behavior in mice locally injected into the BLA with either saline or norBNI (2.5 µg/side). Following injection and recovery, mice were exposed to a single acute swim stress (5 min), placed back in their home cage for 30 min and then assessed in the EPM. As expected, swim stress induced a significant decrease in the percent time in the open arm as compared to saline-injected, non-stressed control animals (Fig 5*C*). The anxiety-like response to acute stress was significantly blocked in mice injected with norBNI in the BLA (Fig. 5*C*) (n = 5–6, Two-way ANOVA; main effect of stress, F _(1,16)_ = 23.54; main effect of treatment, F _(1,16)_ = 5.387), and norBNI alone had no effect on EPM behavior in the absence of stress (Fig 5C). These data further strengthen the conclusion that stress-induces KOR activation in the BLA to mediate acute anxiety-like behaviors.

## Discussion

The principal findings of this study are that stress and CRF-induce dynorphin/KOR activation in the basolateral amygdala to increase anxiety-like behavior through CRF_1_-R activation. Imaging with phospho-KOR antibody revealed that CRF induces dynorphin release and KOR activation in the basolateral amygdala, a brain structure associated with the control of anxiety responses and negative affective state. Previous studies have demonstrated that the CRF system is a critical mediator of depression, drug-seeking, and drug withdrawal behaviors [4], [13], [16], [37], [38]. In addition, several reports have implicated the CRF system in mediating anxiety-like behaviors [10]–[12], [39]. It has been also been suggested that CRF_1_-R-induced anxiety-like behavior may be a critical factor for the initiation of stress-induced reinstatement to drug seeking because the animal may act to re-engage drug seeking as a means to relieve the anxiety and/or dysphoria associated with stress-induced CRF release. Similarly, dynorphin/KOR activity has been demonstrated to be prodepressant, anxiogenic, and mediate stress-induced reinstatement [17]–[20]. There are only a few studies suggesting that CRF-R activation can directly evoke dynorphin release, and these reports established the potential for these two systems to interact *in vivo* [1], [40], [41]. This body of prior work, combined with the present study, reveal a connection between CRF_1_-R dependent activation and the BLA dynorphin/KOR system, and suggest that neurobiological mechanisms for stress-induced anxiety and reinstatement of drug seeking may be mediated via a common CRF-induced dynorphin/KOR dependent pathway.

We used the elevated plus maze (EPM) as a means to model anxiety-like behavior in mice in this study. This assay allows assessment of the behavioral state of a mouse as it explores a dark, closed environment as opposed to a dimly lit, open one. We operationally use the term ‘anxiety’ to describe the increase in percent time spent in a dark closed arm of the EPM, although the actual mood state of the mouse cannot be directly determined. Nevertheless, the EPM is accepted to have predictive validity for pharmacological agents that reduce anxiety in humans [26], [42]. In this report we established conditions where the saline treated animal would spend nearly equal time exploring both open and closed arms of the maze. We presume that the differences in basal anxiety state in our paradigm compared with those of others [11], [22], [32] can be attributed to how intrinsically stressful the assay conditions are and to rodent strain differences. In fact, when we tested our mice under conditions where the ambient light was much brighter, we saw a decrease in the open arm time as predicted. Consistent with prior reports [22], [32], we also found that norBNI pretreatment and dynorphin knockout blocked this effect. It is also noteworthy that differences in results obtained by different research groups concerning the role of CRF_2_-R in anxiety-like behavior has been attributed to genetic background differences, for example, between mixed 129:C57Bl/6 CRF_2_-R knockout animals [11] and as compared to those which were on a more homogeneous C57Bl/6 background [43]. In our study, all knockout mice were backcrossed C57Bl/6 background to facilitate the analysis of the gene effects; however, it is important to recognize that understanding the contributions of differences gene-gene interactions resulting from differences in genetic background and differences in behavioral assay conditions may affect the outcomes and may potentially reveal new insight about factors controlling affective state.

Our finding that CRF_2_-R knockout animals do not develop CRF-induced conditioned place aversion is consistent with our prior work showing that anti-sauvagine (ASV-30), the selective CRF_2_-R antagonist, blocks CRF-induced place aversion. These results were surprising given that CRF_1_-R activation contributes to the aversion evident during drug withdrawal and during reinstatement to drug seeking [3], [6]. Furthermore, CRF_1_-R activity has been demonstrated to be required for the maintenance of ethanol self-administration and mediate withdrawal in nicotine-dependent rats [44], [45]. The difference may suggest that exposure to drugs of abuse may shift the stress response from being principally CRF_2_-R to CRF_1_-R mediated. In addition, KOR activation has also been linked to reinstatement of both drug and alcohol seeking [4]. Finally, it is worth pointing out that the elevated plus maze measures an acute behavioral response, whereas the conditioned place aversion assay probes associative learning with contextual cues. These two behavioral measures are also likely to explain the divergence in the CRF receptor subtype contributing to each dynorphin/KOR-mediated response.

We found that the KORp-ir increased in BLA following CRF injection. Prior work demonstrated that phosphorylation of KOR is mediated by G-protein coupled receptor kinase 3 following receptor activation by the endogenous agonist dynorphin [30]. The BLA is a critical brain structure required for stress, fear and anxiety-like behaviors [36], [46]. In addition, CRF_1_-R has been shown to be expressed in high density within the BLA [47], [48]. Additionally, CRF_1_-R induces excitatory activity in the BLA and has been demonstrated to be required for the modulation neuronal excitatory potentials in BLA [49]. Together with anatomical and behavioral results revealing a role for CRF_1_-R activity in the BLA, a role for these neurons is implicated in modulating anxiety responses and aversive memories including the modulation of hedonic state [4], [50]. We previously showed that ASV-30 (R2 antagonist) blocked the increase in KORp-ir in the BLA [1], thus we were surprised to see a lack CRF-induced KORp-ir in the BLA of CRF_1_-R (−/−) mice and that the CRF_1_-R-selective antagonism by antalarmin attenuated KORp-ir in the BLA. However, it is likely that CRF-induced dynorphin release in the BLA converges to initiate two separate behavioral responses via KOR activation. In finding that KORp-ir was not increased in CRF_1_-R (−/−) animals or in CRF_1_-R antagonist-pretreated mice following CRF treatment, combined with a lack of a CRF-induced or stress-induced increase in the percent open arm time in mice locally injected with norBNI into the BLA, we infer that stress mobilizes CRF to induce dynorphin release in BLA via CRF_1_-R activation.

We were particularly surprised to find that CRF-injection in *CRF_2_-R* knockout mice produced conditioned place preference, as opposed to solely blocking CRF-induced place aversion. These data are remarkably consistent with our previous report in dynorphin knockout animals where CRF-induced place preference [1]. The reasons for the apparent rewarding effects of CRF in CRF_2_R knockout mice are unknown, but prior studies have shown that CRF_1_-R activation can increase dopamine cell firing and dopamine release in brain structures associated with reward, including the ventral tegmental area and nucleus accumbens [5], [35]. Similarly, CRF-induced a significant increase in the time spent in the open arm in *pDyn* knockout mice, further corroborating the conceptual framework that in the absence of dynorphin, mice may be less apprehensive and more likely to explore the open arm of the elevated plus maze. These results are in conflict with the lack of place preference to nominally selective CRF_1_-R agonists in wildtype mice. Thus, further study of drug selectivity and possible compensatory change in response to *CRF_2_-R* or *pDyn* gene deletion is required.

Recent work has suggested that KOR activation can mediate anxiety-like behavior [22] and that prodynorphin-derived peptides can regulate basal anxiety behavior [32]. In contrast, other studies have reported that the dynorphin/KOR system may act to decrease anxiety-like behavior [51], [52]. Additionally, it has been reported that kappa opioid receptor knockout animals showed similar percent time spent in open arms to their wild type counterparts [53]. Studies measuring the effect of KOR-agonists on acute behavior and reports implicating the utility of KOR agonists as therapeutic agents must be interpreted with caution since KOR agonism is thought to be hallucinogenic [54], dysphoric [1], [55], and can induce hypolocomotor activity [53]. The results of the present study along with others [17], [21]–[23], [32] further support recent efforts to develop and explore KOR antagonists as therapeutic agents for the treatment of anxiety and depression-related diseases.

The discovery of CRF was a major milestone, and the development of CRF receptor antagonists was a big step forward, but CRF is essential for a healthy response to stress and inhibiting CRF receptors broadly may block both the adverse effects and the protective effects of CRF necessary for survival. The converging effects of stress on the dynorphin kappa opioid system in the BLA and its key role in mediating the adverse effects of stress implies that new therapeutic strategies should be directed toward the development of KOR antagonists.

## Supporting Information

Figure S1 Anxiety-like behavior is mediated by the dynorphin/KOR system. Brightly lit conditions produced a significant anxiety-like effect (decrease in % open arm time) in the elevated plus maze (EPM) compared saline controls. This anxiety-like effect was blocked by pretreatment with the KOR antagonist norBNI (10 mg/kg, i.p., 2.5 hr prior to test) (n = 4–6; p<0.05; t-test, t6 = 2.917) Dyn(+/+) wild type littermates showed a significant anxiety-like response, which was not evident in prodynorphin knockout animals (Dyn −/−) (n = 5; p<0.05, t-test, t7 = 3.147).
(0.56 MB TIF)

Figure S2 The Ventral posteromedial thalamic nucleus (VPN) expresses Kappa-Opioid receptors. Representative image of U50, 488 challenged (10 mg/kg, i.p., 30 min) BLA brain section stained with KORp antibody. Data confirms that this thalamic nucleui expresses KOR.
(2.74 MB TIF)
