# Supplementary figures and images for: CRF1-R Activation of the Dynorphin/Kappa Opioid System in the Mouse Basolateral Amygdala Mediates Anxiety-Like Behavior
Source: PLoS One. 2009 Dec 31;4(12):e8528. doi: 10.1371/journal.pone.0008528 (PMC2795205; doi:10.1371/journal.pone.0008528)

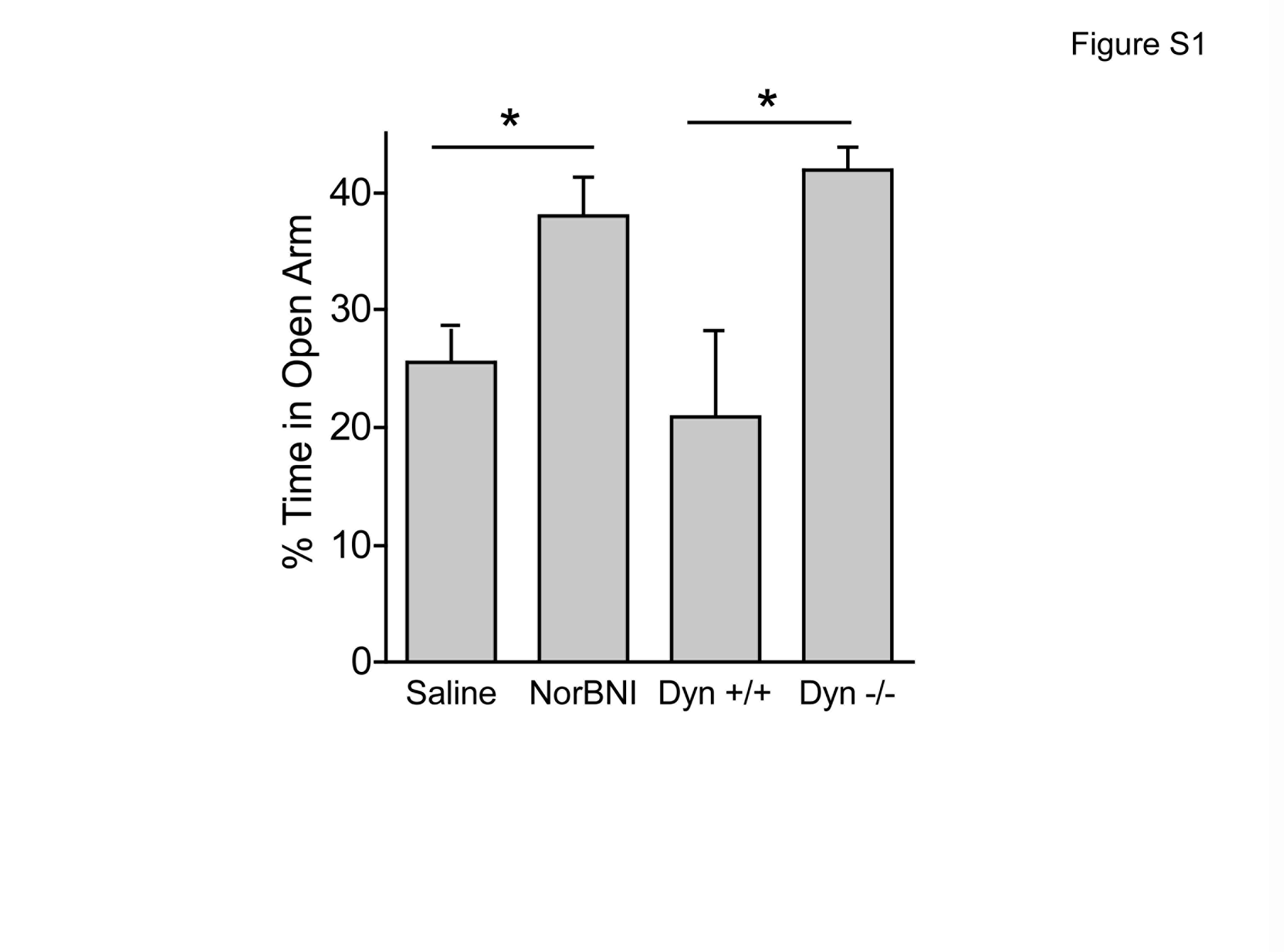

Supplement: Figure S1 — Anxiety-like behavior is mediated by the dynorphin/KOR system. Brightly lit conditions produced a significant anxiety-like effect (decrease in % open arm time) in the elevated plus maze (EPM) compared saline controls. This anxiety-like effect was blocked by pretreatment with the KOR antagonist norBNI (10 mg/kg, i.p., 2.5 hr prior to test) (n = 4–6; p<0.05; t-test, t6 = 2.917) Dyn(+/+) wild type littermates showed a significant anxiety-like response, which was not evident in prodynorphin knockout animals (Dyn −/−) (n = 5; p<0.05, t-test, t7 = 3.147). (0.56 MB TIF) [file pone.0008528.s001.tif]

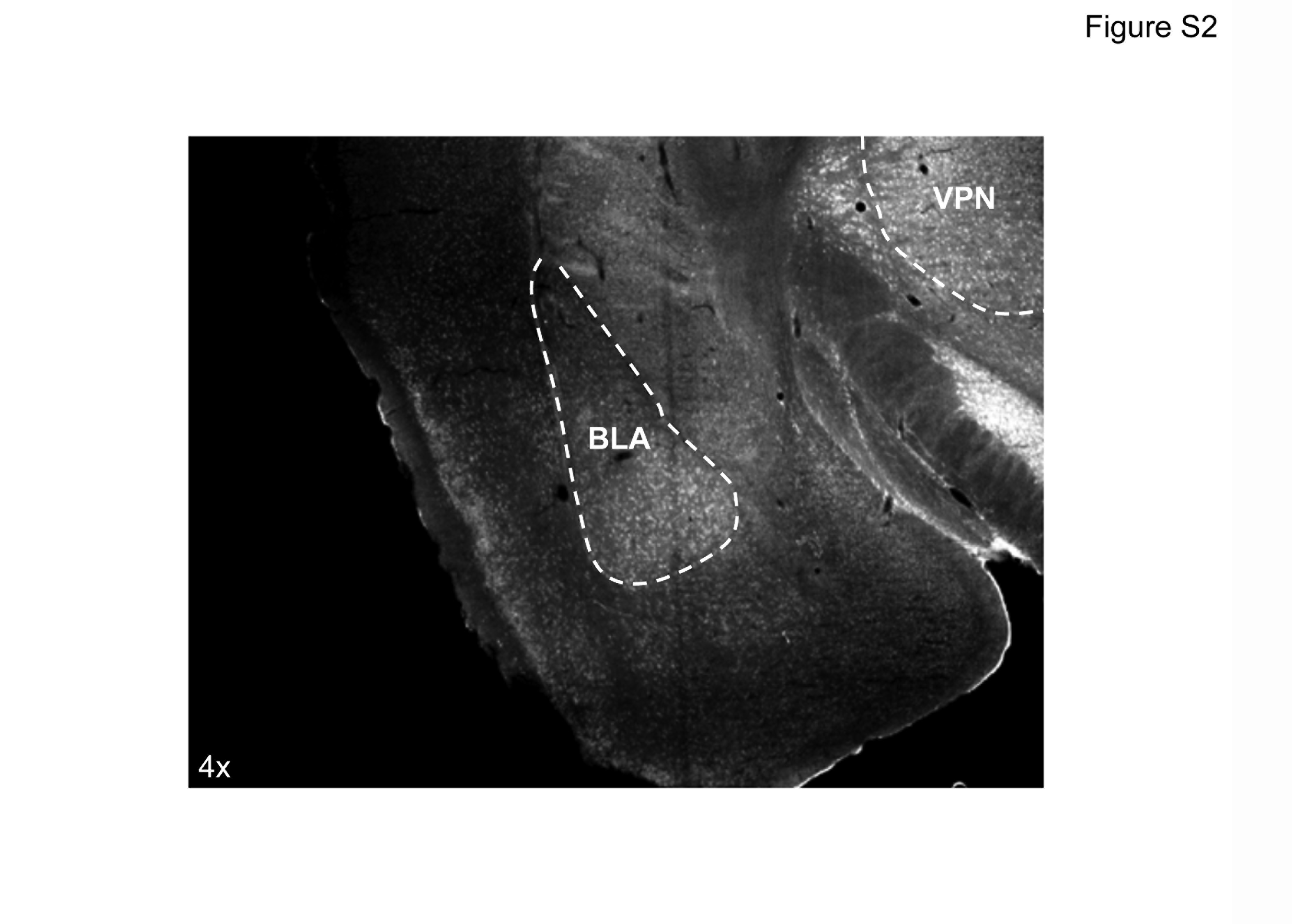

Supplement: Figure S2 — The Ventral posteromedial thalamic nucleus (VPN) expresses Kappa-Opioid receptors. Representative image of U50, 488 challenged (10 mg/kg, i.p., 30 min) BLA brain section stained with KORp antibody. Data confirms that this thalamic nucleui expresses KOR. (2.74 MB TIF) [file pone.0008528.s002.tif]
